# Extraconal Orbital Soft Tissue Metastasis Secondary to Prostate Cancer: An Unusual Presentation

**DOI:** 10.14740/wjon807w

**Published:** 2014-06-25

**Authors:** Sakshi Kapur, Han Xiao

**Affiliations:** a Department of Internal Medicine, Overlook Medical Center, 99 Beauvoir Ave, Summit, New Jersey, 07902, USA; b Division of Medical Oncology, Memorial Sloan-Kettering Cancer Center Basking Ridge, 136 Mountain View Boulevard, Basking Ridge, New Jersey, 07920, USA

**Keywords:** Extraconal orbital metastasis, Androgen blockade therapy, Choroidal metastasis

## Abstract

Prostate cancer is mostly known to metastasize to the bony skeleton. Soft tissue metastasis involving visceral organs such as the liver, lung and brain are unusual and rare manifestations of this cancer. However, with better treatment modalities and increased survival, the incidence of these unusual presentations, seems to have risen in the last few years. Prostate cancer is rarely known to metastasize to the orbit. Although cases of bony metastasis involving the orbit have been reported, soft tissue metastasis involving the orbit is a very rare presentation. Most soft tissue metastasis to the orbit involves the uveal tract, with prostate cancer being the most common primary cancer to metastasize to the iris (uveal tract). Our paper highlights a case of extraconal orbital soft tissue metastasis secondary to prostate cancer, an extremely rare presentation. Patient was started on combined androgen blockade therapy. Three-month repeat MRI orbit showed that the extraconal orbital mass had decreased in size significantly. His clinical symptoms have resolved at the time of this report. To our knowledge, this is the first reported case of its kind.

## Introduction

Prostate cancer (PC) is the most common non-skin cancer in men [1]. It is the second most leading cause of cancer death in the United States. In 2011, 240,890 men were diagnosed with PC and 33,730 died of the same [1]. PC is mostly known to metastasize to the bony skeleton [2]. Soft tissue metastasis involving visceral organs such as the liver, lung and brain are unusual manifestations of this cancer. The lack of skeletal metastasis does not exclude the possibility of visceral/distant metastasis, and serum PSA levels usually don not correlate with the extent of metastatic disease [3].

## Case Report

A 79-year-old male presented to an ophthalmologist’s outpatient office with progressively worsening vision in his right eye over 3 - 4 months. He also complained of diplopia and proptosis in the same eye. Review of systems was positive for increased urinary frequency and occasional nocturia over the last 6 months. However, he denied any incontinence, dysuria, flank pain or fever or chills.

Physical examination revealed a well-built man with no acute distress. Vital signs were within normal limits, except for an elevated blood pressure of 146/87 mm Hg. Head and neck exam was positive for mild pallor; however, no icterus or lymphadenopathy was noted. Rest of the systemic exam was unremarkable. His karnofsky performance status scale was 80%. Ophthalmological exam revealed visual acuity of 20/30 and 20/25 in right and left eye, respectively. Intra-ocular pressures were 13 mm Hg and 10 mm Hg in right and left eye, respectively. Proptosis of 5 mm and a 60% reduction in all fields of right eye were noted. Examination of the left eye revealed full motility, no proptosis and 2+ nuclear sclerotic lens changes. Dilated fundus exam was unremarkable in both eyes.

**Figure 1:**
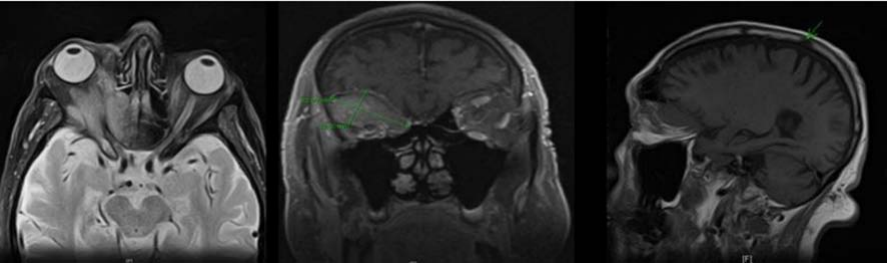
MRI of the head showing a right intra-orbital mass measuring 4.3 × 2.3 cm, extending into the anterior cranial fossa (superiorly); lamina papyracea, posterior ethmoid sinus and anterior lateral sphenoid sinus (medially); and posterior lateral orbital wall and sphenoid wing (laterally); sclerotic lesion in the right parietal bone also noted.

Magnetic resonance imaging (MRI) of the head revealed a hypointense, mildly enhancing, multi-compartmental soft tissue mass lesion in the superior right extraconal space measuring approximately 4.3 × 2.3 cm. This mass was seen extending and breaking through the orbital roof into the anterior cranial fossa superiorly, and medially extending into the lamina papyracea, posterior ethmoid sinus and the anterior lateral sphenoid sinus. Lateral extension into the posterior lateral orbital wall and sphenoid wing was noted. This mass was also seen wrapping and compressing the optic nerve sheath complex in the posterior intraconal space at the orbital apex. A 1.2 cm sclerotic lesion in the right parietal bone was also noted (Fig. 1). Patient underwent a right orbitotomy, and the biopsy of the orbital mass revealed a poorly differentiated adenocarcinoma.

**Figure 2:**
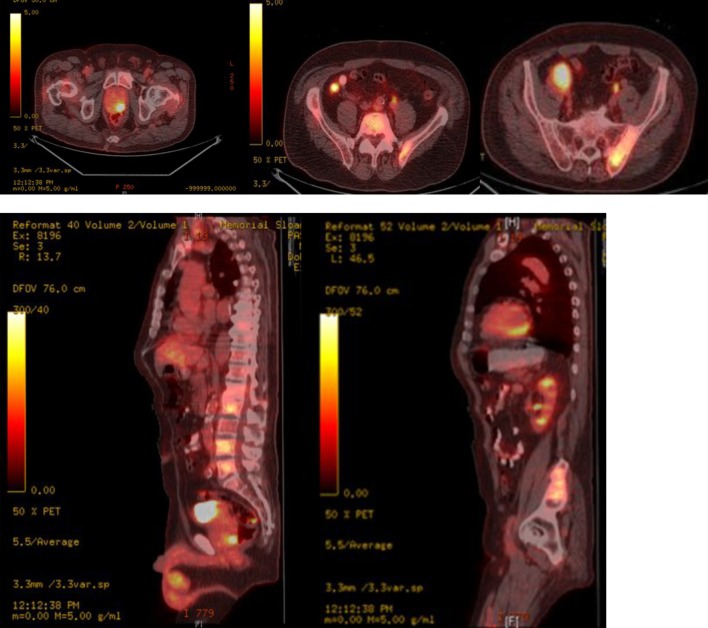
Whole body PET-CT showing FDG avid uptake in posterior lobe of prostate, axial spine, iliac bones and bilateral iliac lymph nodes.

A thorough systemic evaluation was performed to look for a primary source of malignancy. Laboratory work-up revealed a normal complete blood count and comprehensive metabolic panel except for an elevated alkaline phosphatase level of 317 units/L (normal range: 49 - 129 units/L). Whole body PET-CT showed: an FDG avid mass in the right posterior orbit extending into the ethmoidal cells (SUV 4.2), FDG avid focus in the left posterior aspect of the prostate gland suspicious of a malignancy (SUV 8.9), bilateral FDG avid enlarged iliac nodes (SUV 6.1 - 6.3) and multiple FDG avid sclerotic bony lesions, suspicious of metastasis (Fig. 2).

**Figure 3:**
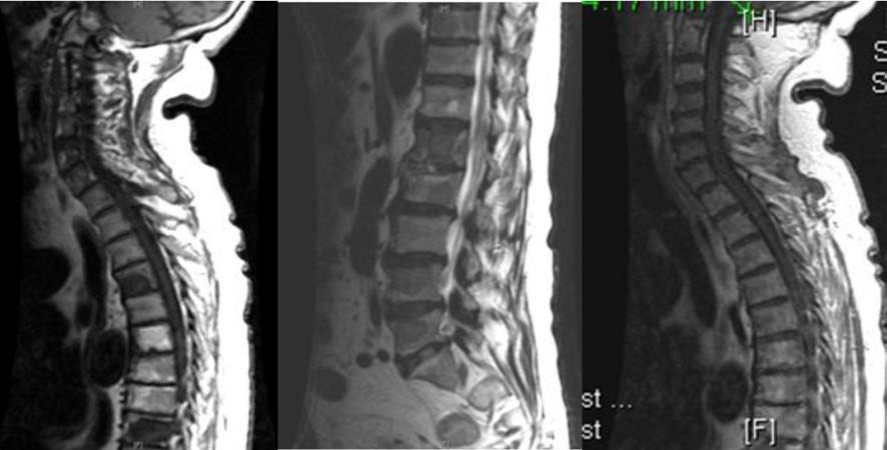
MRI of the spine showing multi-level osseous metastatic infiltration throughout the spinal axis particularly involving the lower thoracic and lumbar regions.

Further work-up revealed an elevated prostate specific antigen (PSA) and acid phosphatase level of 521.20 ng/mL (normal range: 0 - 4 ng/mL) and 70.2 ng/mL (normal range: < 1.5 ng/mL), respectively. A urological consultation was obtained, and prostate biopsy results revealed a poorly differentiated adenocarcinoma with a Gleason score (GS) of 8 and 9 in 11 out of 12 cores, similar to the orbital lesion. Immunohistochemical stains on the orbital tissue (biopsy) were positive for PSA, PSMA, AE1:AE3 and Cam5.2 cytokeratins, and negative for TTF-1, CDX2, CK7, CK20 and EMA. MRI of the spine confirmed multi-level osseous metastatic infiltration throughout the spinal axis particularly involving the lower thoracic and lumbar spine (Fig. 3).

This confirmed the diagnosis of adenocarcinoma arising in the prostate gland, with metastasis to the soft tissue of the right eye (stage IV). Patient was started on bicalutamide and degarelix (combined androgen blockade therapy). Three-month repeat MRI orbit showed that the extraconal orbital mass had decreased in size significantly. Eight months later patient continues to follow up as an outpatient, and his vision and diplopia have improved significantly.

## Discussion

Adenocarcinoma of the prostate accounts for 3.6-4% of all orbital metastasis [4]. PC can spread to the orbit by either hematogenous or venous route. Hematogeous spread involves the ophthalmic branch of the internal carotid artery, whereas venous spread is through the Bateson’s venous plexus and the cranial venous sinuses (ophthalmic vein). However, in a study by Shields et al out of the 100 patients with orbital metastasis, 12% of the patients had a prostate primary [5]. In another study by Goldberg et al, the mean age at diagnosis was 70 years [6]. The prognosis, once orbital metastasis occurs, has been reported as 4 - 26 months [7, 8]. In general, patients present with ocular symptoms such as decreased vision, diplopia, proptosis and periorbital edema. Metastasis to the bony orbit although rare, has been reported in the past [9, 10]. Rarely, PC has been reported to metastasize to the optic canal and the pituitary region [11, 12].

However, soft tissue metastasis to the orbit is extremely rare. Most cases of soft tissue orbital metastasis secondary to PC involve the uveal tract [13-17] (Table 1). PC is the most common primary cancer to metastasize to the iris (uveal tract) and the reported incidence ranges from 8% to 10% [18, 19]. Leonard et al reported a rare case of a 51-year-old male who developed diplopia secondary to metastatic infiltration of the lateral rectus muscle [20].

**Table 1:** Case Reports of Uveal Tract Metastasis Secondary to Prostate Cancer

Author	Age (years)	Year	Site of orbital metastasis
Sarenac et al [13]	69	2012	Bilateral iris metastasis
Keizur et al [14]	65	1995	Choroidal metastasis
Liu et al [15]	60	1992	Combined scleroidal and choroidal metastasis
Dieckert et al [16]	54	1982	Choroidal metastasis
Zappia et al [17]	58	1972	Metastasis to both the choroid and optic nerve, mimicking papilledema

**Figure 4:**
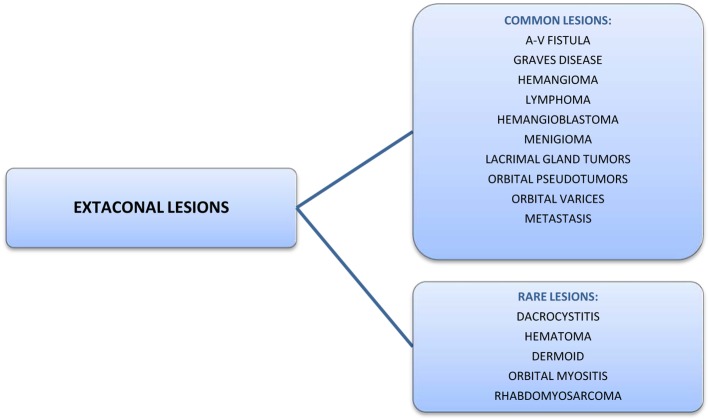
Lesions arising in the extraconal space.

Extraconal space is the area outside muscle cone in the eye. Various lesions are known to arise in this space (Fig. 4). Although both bony and soft tissue metastasis involving the uveal tract have been reported in the past, extraconal orbital soft tissue metastasis secondary to PC is not known. Our case is unusual because: first, the patient presented with orbital metastasis as the first manifestation of PC and second, patient developed a soft tissue metastatic lesion in the orbit, arising in the right superior extraconal space, making it an extremely rare presentation. Autorino et al reported a rare case of a 73-year-old male with ocular metastasis as the first indication of PC [21].

Treatment options for orbital metastasis secondary to PC mainly include androgen blockade therapy and cranial irradiation. Cases of complete regression of choroidal metastasis following hormonal manipulation in patients with PC have been reported [14, 22]. Radio-therapy includes both external beam radiation and plaque radio-therapy. Dobrowsky et al in their study reported a 50% complete and 35% partial response following irradiation, in patients with choroidal metastasis from various cancers, including prostate [23]. However, irradiation is mainly reserved for patients who fail hormonal therapy. Lastly, enucleation although rarely used, is indicated in patients with ocular pain and complete loss of vision.

### Conclusion

Extraconal orbital soft tissue metastasis secondary to prostate cancer has not been reported in the past. To our knowledge, this is the first reported case of extraconal soft tissue metastasis secondary to PC.
